# Paired analysis of flexible and navigable suction ureteral access sheath vs. conventional ureteral access sheath, both combined with needle-perc assisted endoscopic surgery, for the treatment of <2 cm lower calyceal stones with unfavorable anatomy

**DOI:** 10.3389/fsurg.2025.1586882

**Published:** 2025-07-22

**Authors:** Daxun Luo, Zheng Xu, Tianfu Ding, Yubao Liu, Jianxing Li

**Affiliations:** Department of Urology, Beijing Tsinghua Changgung Hospital, Research Center for Urinary Disease, School of Clinical Medicine, Tsinghua University, Beijing, China

**Keywords:** FANS, ureteral access sheath, ureteroscopy, needle-perc assisted endoscopic surgery, kidney stones

## Abstract

**Purpose:**

To compare the flexible and navigable suction ureteral access sheath (FANS) with the conventional ureteral access sheath, both in combination with Needle—perc Assisted Endoscopic Surgery (NAES), for treating <2 cm lower calyceal stones with unfavorable anatomy.

**Materials and methods:**

Data of patients admitted to Beijing Tsinghua Changgung Hospital with <2 cm stones with unfavorable anatomy of the renal lower calyx from August 2023 to May 2024 were collected retrospectively, and matched parameters such as age, gender, BMI, stone size, CT values, laboratory tests, and anatomical features of the lower calyces of the kidney were recorded. Both groups of patients were treated with NAES, and patients who were treated with FANS were compared with those who received a conventional ureteral access sheath in a pairwise analysis (1:1). Data were analyzed using *t*-test, Mann–Whitney *U*-test, and chi-square test.

**Results:**

Both groups had similar baseline characteristics. The immediate stone-free rate (SFR) was better in the FANS group than in the conventional ureteral access sheath treatment group (88% vs. 64%, *p* = 0.044). The duration of surgery was shorter in the former than in the latter in both groups (100.75 ± 25.32 min vs. 116.21 ± 35.56 min, *p* = 0.048). No statistically significant differences were observed between the two groups in postoperative ESWL treatment, postoperative creatinine, hospital stay, 1-month SFR, and complication rates.

**Conclusions:**

In the NAES procedure, compared with conventional ureteral access sheath, the FANS ensures safety while also demonstrating greater effectiveness for treating kidney stones in patients with unfavorable renal lower calyx anatomy of less than 2 cm.

## Introduction

Due to changes in modern lifestyle (sedentary habits, less water intake) and environmental factors, the incidence of kidney stones is increasing year by year. Current treatments for urinary tract stones are diverse, including drug expulsion, extracorporeal shock wave lithotripsy (ESWL), retrograde intrarenal surgery (RIRS), and percutaneous nephrolithotomy (PCNL) (1). RIRS is recommended as one of the first-line treatments for renal stones <2 cm due to its minimal invasiveness and rapid recovery, but it has limitations, such as the inability to remove stones when the renal lower calyx anatomy is unfavorable. Consequently, improving the SFR for patients with anatomical abnormalities of the renal lower calyx and a small stone burden (<2 cm) has become a significant research focus. In recent years, the development of Needle-perc and the FANS has significantly improved the efficiency of surgical stone fragmentation, offering better treatment options for the aforementioned patients (2). Needle-perc has a thin channel and minimal damage, with a high puncture success rate under ultrasound guidance, and its clinical application has increased. FANS can reduce renal pressures and aspirate powdered stones, improving the immediate postoperative SFR. This study compares the therapeutic effects and safety of Needle-perc combined with RIRS for unfavorable anatomy of lower calyx stones when using either FANS or a conventional ureteral access sheath.

## Methods

### Data collection

**Figure 1:**
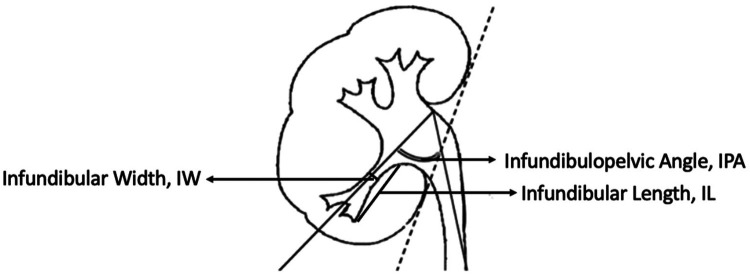
Illustration of a kidney highlighting measurements. Infundibular Width (IW), Infundibular Length (IL), and Infundibulopelvic Angle (IPA) are marked with arrows, showing positions in renal anatomy.

**Figure 2:**
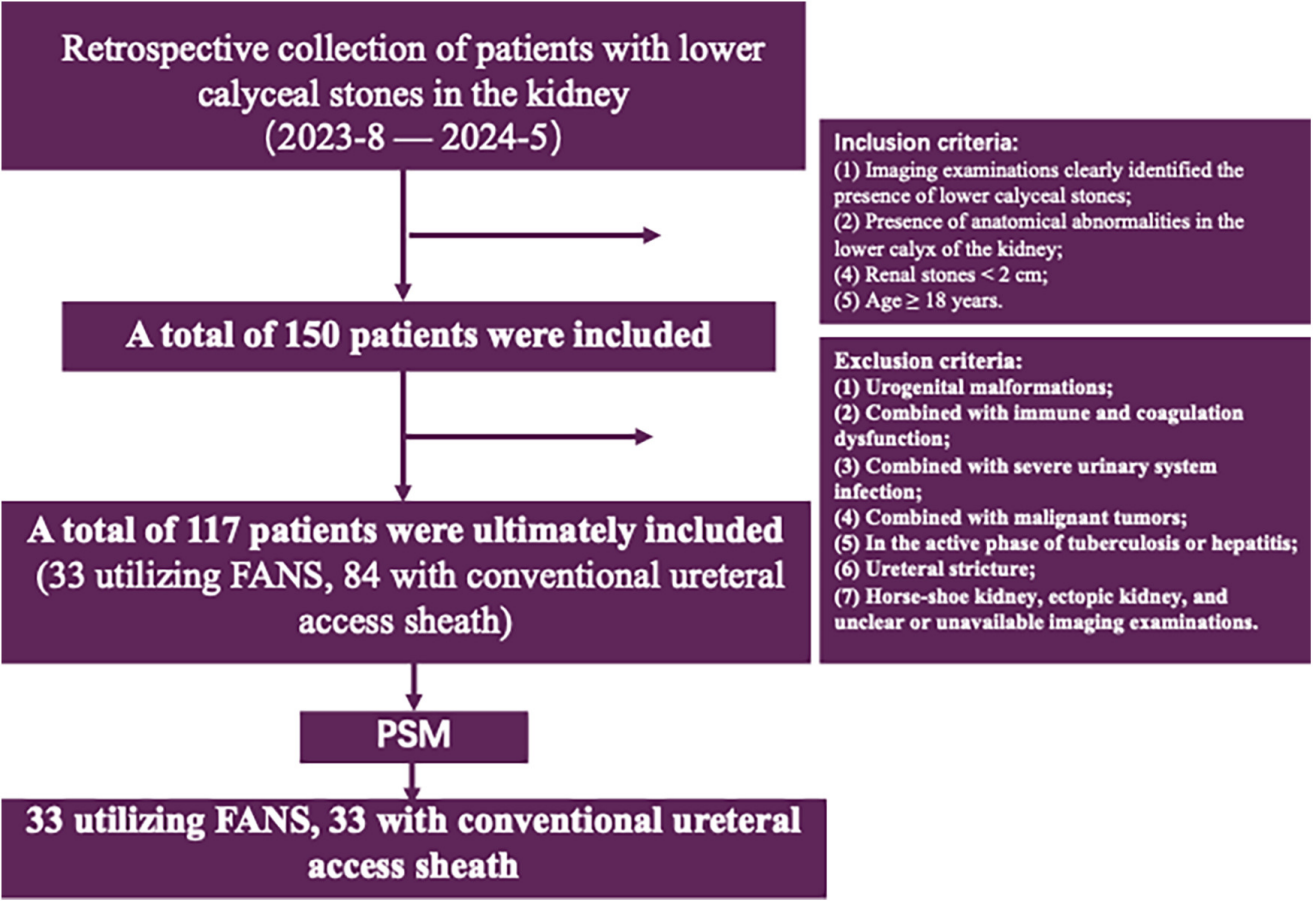
Flowchart detailing a retrospective study of patients with lower calyceal stones in the kidney from August 2023 to May 2024. It began with 150 patients, narrowing to 117 after applying inclusion criteria like age over 18 and stone size under 2 centimeters. Exclusions included urogenital malformations and severe infections. Ultimately, two groups of 33 patients each were formed, based on the use of FANS or conventional ureteral access sheath, after applying propensity score matching (PSM).

This retrospective study collected clinical data from patients with lower calyceal stones smaller than 2 cm lower calyceal stones and unfavorable anatomical conditions, who were treated at the Department of Urology, Beijing Tsinghua Changgung Hospital, between August 2023 and May 2024. The following information was collected for each patient: demographic details, renal and stone imaging data, including age, sex, BMI, stone size, stone burden, stone CT value, and laboratory test results. All patients underwent routine preoperative assessments, including laboratory tests, imaging studies, and urine cultures. Antibiotics were administered to patients with urinary tract infections prior to surgery. Stone size was defined as the maximum diameter of the stone on computed tomography (CT) images. The anatomical parameters obtained the infundibulopelvic angle (IPA), infundibular length (IL), and infundibular width (IW) (Figure 1). Anatomical parameters of the lower calyx were measured using three-dimensional (3D) CT reconstruction. Reasons for choosing 3D reconstruction to measure IPA, IL, and IW: (1) Offers comprehensive anatomical details and clear visualization of complex structures in relation to surrounding tissues. (2) Reduces measurement errors by avoiding structural overlap and image distortion present in 2D CT and IVU. (3) Enhances measurement accuracy with no magnification errors, utilizing multiplanar reconstruction and various imaging techniques. (4) Provides intuitive visualizations that aid in preoperative planning and postoperative analysis, helping doctors make more accurate diagnostic and treatment decisions. Unfavorable anatomy of the lower renal calyx (3–5) was defined as meeting at least one of the following criteria: IPA < 30°, IL ≥ 3 cm, or IW < 5 mm. A total of 117 patients who met the above criteria were included in the study. Among them, 33 patients underwent treatment with the FANS technique, while 84 received treatment with a conventional ureteral access sheath. Stone fragments measuring ≤ 2 mm in diameter were considered stone free. Postoperative complications were evaluated according to the Clavien-Dindo classification system. Inclusion Criteria: (1) Imaging studies (CT, renal ultrasound, intravenous pyelography) confirming the presence of lower calyceal stones. (2) Unfavorable lower calyceal anatomy. (3) Kidney stones < 2 cm in diameter. (4) Age ≥18 years. Exclusion Criteria: (1) Urinary tract malformations. (2) Immunological or coagulation disorders. (3) Severe urinary tract infections. (4) Malignancies. (5) Active tuberculosis or hepatitis. (6) Ureteral strictures. (7) Horseshoe kidney, ectopic kidney, or unclear/unusable imaging results. Patient enrollment algorithm is illustrated in Figure 2. This study was approved by the Ethics Committee of Beijing Tsinghua Changgung Hospital (Review Number: 24747-0-02).

### Surgical methods

In this study, all surgeries in this study were performed by designated, experienced surgeons at our center (each performing ≥100 urinary stone surgeries annually). The surgical team employed standardized surgical instruments and parameters, such as an irrigation pressure of 0.1 MPa and an aspiration flow rate of 100 ml/min. The maximum suction pressure of the FANS was set at −0.04 MPa to maintain renal pressure balance. Preoperative CT—based multidisciplinary planning was used for planning access and scope insertion. Two groups of patients with lower calyceal stones and unfavorable anatomy were treated using RIRS combined with Needle-perc. In the first group, an 8F ureteroscope and a 12/14F FANS were used. The holmium laser [200 um holmium laser ultra-fine fiber (6), from Swiss company EMS] was set to long pulse width with an energy of 8–10W for stone fragmentation, and stone fragments were aspirated through the sheath. After the procedure, a double-J stent was placed and scheduled for removal 2–4 weeks postoperatively. The second group followed a similar approach but used a conventional ureteral access sheath, with stone fragments retrieved using a stone basket. Both groups underwent postoperative imaging within 24 h to evaluate the immediate SFR.

The Needle-perc is an advanced minimally invasive urological instrument developed by Professor Jianxing Li's team at the Department of Urology, Beijing Tsinghua Changgung Hospital, Tsinghua University. The instrument has an outer diameter of 4.2 Fr, an inner diameter of the working channel of 3.6 Fr, and a length of 152 mm. It is currently the smallest diameter nephroscope in the world and is equipped with capabilities for puncture, visualization, and stone fragmentation. The needle-perc contains three channels: a visualization fiber, a laser fiber, and an irrigation port.

### Statistical methods

This retrospective study used SPSS statistical software version 27.0 for data organization, analysis, and summary. Propensity score matching (PSM) was applied to minimize baseline differences between the conventional ureteral access sheath group and the FANS group. Variables such as age, sex, BMI, stone burden, CT values, and lower calyceal anatomical parameters were included in a 1:1 nearest neighbor matching between the two groups. Post matching, we calculated the standardized mean difference (SMD) to check the matching quality. An SMD below 0.5 indicates good balance, permitting subsequent analyses to proceed. Normality of the continuous variables was assessed using the normality test (*p* > 0.05). Variables with a normal distribution were compared using the independent *t*-tests, while categorical variables were analyzed using the chi-square test. For variables with a non-normal distribution, the Mann–Whitney *U*-test was applied. A *p*-value < 0.05 was considered statistically significant. The stone burden was calculated using the following surface area formula:

Stone burden = maximum diameter × width × π × 1/4 (7)

### Clinical follow-up

All patients underwent routine follow-up at the outpatient 1 month after surgery. Stone-free status was defined as the presence of residual fragments measuring ≤2 mm. Based on previous experience, cystine and uric acid stones are difficult to detect via KUB/ultrasound. In this study, on the first postoperative day, we performed CT scans on patients to determine the immediate SFR. At one-month follow-up, we conducted KUB or CT scans on the patients to determine the final SFR. Postoperative ESWL therapy for stone expulsion was considered an auxiliary treatment. Perioperative complications were recorded and graded using the modified Clavien-Dindo classification system to assess their severity during and after surgery.

## Results

Pre-matching baseline data for the two patient groups are presented in Table 1. After matching, the baseline demographic and clinical characteristics between the two groups were comparable (Table 2). Intraoperative and postoperative outcomes were compared in Tables 3, 4. The operative time in the FANS group was significantly shorter than in the conventional ureteral access sheath group, with statistical significance (100.75 ± 25.32 min vs. 116.21 ± 35.56 min, *P* = 0.048). The immediate SFR in the FANS group was significantly higher compared to the conventional ureteral access sheath group (88% vs. 64%, *P* = 0.044, 95%CI = 1.17–14.65). Both groups showed elevated serum creatinine levels on the first postoperative day. The intraoperative blood loss in the FANS group was lower than in the conventional ureteral access sheath group (5.91 ± 4.22 ml vs. 6.82 ± 7.57 ml), but this difference was not statistically significant (*P* = 0.549). One patient in the FANS group and four patients in the conventional ureteral access sheath group underwent postoperative ESWL therapy as an auxiliary treatment, with no significant difference between the groups. At the 1-month follow-up, the SFR in the FANS group remained higher than that in the conventional ureteral access sheath group, but the difference was not statistically significant (97% vs. 88%, *P* = 0.352). One complication observed in the FANS group (Fever in 1 case), while two complications occurred in the conventional ureteral access sheath group (Hemorrhage in 1 case, pulmonary embolism in 1 case), with no statistically significant difference (3% vs. 6%, *P* = 1.0). One patient in the conventional ureteral access sheath group developed a postoperative pulmonary embolism, which was successfully treated with low-molecular-weight heparin and rivaroxaban. No Grade III-IV complications were observed in either group in this study (8).

**Table 1:** Pre-matching baseline data.

Parameters	FANS (N + R)	Conventional ureteral access sheath (N + R)	*P*-value
Number of people, n, %	33 (28.2%)	84 (71.8%)	
sex, n, %			0.49
Female	12	25	
Male	21	59	
Age, y, mean ± SD	50.64 ± 13.93	53.92 ± 14.28	0.26
Height, m, mean ± SD	167.76 ± 8.67	168.17 ± 14.68	0.88
Weight, kg, mean ± SD	70.74 ± 14.56	73.89 ± 16.04	0.33
BMI, kg/m2, mean ± SD	24.95 ± 3.77	30.06 ± 4.27	0.51
Stone area, mm^2,^ mean ± SD	56.89 ± 29.94	54.48 ± 37.25	0.74
CT values, Hu, mean ± SD	758.58 ± 336.20	784 ± 326.66	0.70
Lower-pole anatomic parameters			
IPA (angle), mean ± SD	24.8 ± 2.32	25.24 ± 3.22	< 0.01
IL (cm), mean ± SD	3.27 ± 0.98	2.86 ± 0.61	0.04
IW (cm), media (IQR)	0.52 (0.3)	0.47 (0.27)	0.59

N + R is one of the NAES mode, NAES needle-perc-assisted endoscopic surgery, BMI body mass index, IPA infundibulopelvic angle, IL infundibular length, IW infundibular width.

**Table 2:** Post-matching baseline data.

Parameters	FANS (N + R)	Conventional ureteral access sheath (N + R)	*P*-value	SMD
Number of people, *n*, %	33 (50%)	33 (50%)		
Sex, *n*, %				0.34
Female	12	7		
Male	21	26	0.277	
Age, y, mean ± SD	50.64 ± 13.93	53.42 ± 14.52	0.429	0.20
Height, m, mean ± SD	167.76 ± 8.67	170.18 ± 8.74	0.262	0.28
Weight, kg, mean ± SD	70.74 ± 14.56	75.42 ± 11.98	0.159	0.36
BMI, kg/m^2^, mean ± SD	24.95 ± 3.77	25.97 ± 3.11	0.235	0.30
Stone area, mm^2,^ mean ± SD	56.89 ± 29.94	54.83 ± 40.01	0.813	−0.06
CT values, Hu, mean ± SD	758.58 ± 336.2	780 ± 333.4	0.795	0.07
Lower-pole anatomic parameters				
IPA (angle), mean ± SD	24.8 ± 2.32	25.96 ± 2.75	0.068	0.46
IL (cm), mean ± SD	3.27 ± 0.98	2.92 ± 0.58	0.087	−0.43
IW (cm), media (IQR)	0.52 (0.3)	0.48 (0.245)	0.488	−0.06

**Table 3:** Intraoperative date.

Parameters	FANS (N + R)	Conventional ureteral access sheath (N + R)	*P*-value
Operative duration, min, mean ± SD	100.75 ± 25.32	116.21 ± 35.56	0.048
Intraoperative blood loss, ml, mean ± SD	5.91 ± 4.22	6.82 ± 7.57	0.549

**Table 4:** Postoperative data.

Parameters	FANS (N + R)	Conventional ureteral access sheath (N + R)	95%CI	*P*-value
Hospitalization time, n, day, media（IQR）	3 (2)	3 (1)	[0.341, 0.36]	0.35
Postoperative creatinine, μmoI/L, mean ± SD	88.64 ± 25.56	94.48 ± 41.67	[−0.315, 0.652]	0.495
*Δ* Creatinine, μmoI/L, media (IQR)	2 (11)	3（13）	[0.563, 0.582]	0.57
Complications, n, %				
I、II	1 (3%)	2 (6%)	[0.48 (0.04, 5.62)]	1.0
III、IV				
SFR immediately after surgery, %	88%	64%	[4.14 (1.17, 14.65)]	0.04
SFR 1 month after surgery, %	97%	85%	[5.71 (0.63, 51.89)]	0.20
Auxiliary treatment, n,%	1 (3%)	4 (12.1%)	[0.23 (0.02, 2.15)]	0.36

## Discussion

The treatment methods for renal stones mainly include extracorporeal shock wave lithotripsy (ESWL), retrograde intrarenal surgery (RIRS), and percutaneous nephrolithotomy (PCNL). For patients with lower calyceal stones, PCNL represents a guideline-recommended standard surgical approach. Although PCNL achieves superior stone clearance rates, it is associated with a significant risk of intraoperative hemorrhage (9, 10), which can significantly impact postoperative renal function. Patients are required to carry a drainage tube after surgery. Moreover, due to the absence of significant hydronephrosis in most renal lower calyx stones, percutaneous puncture is challenging, and the channel is easily lost during dilation. Therefore, it is not suitable for stones with a small burden. Previous studies have shown that complications of PCNL are often related to the size of the channel (11–13).

Based on these considerations, our center has innovatively promoted the use of Needle-perc, which is currently the thinnest visual nephroscope available in clinical practice. Building on this, we have proposed Needle-perc Assisted Endoscopic Surgery (NAES), which includes two surgical approaches: the standard percutaneous nephrolithotomy combined with Needle-perc technique (S + N) and the ureteroscopy combined with Needle-perc technique (N + R) (5, 14). For the ureteral access component within the N + R approach, this study introduces the FANS—a novel device featuring active steerable navigation and continuous negative pressure suction (−0.04 MPa). This study focused on patients with lower calyceal stones complicated by unfavorable anatomical features—such as an excessive infundibulopelvic angle (IPA) or elongated infundibular length (IL)—where RIRS monotherapy yields suboptimal clearance rates. Given this limitation, both cohorts received the combined N + R approach, with the sole methodological divergence residing in the ureteral access method during the RIRS phase: experimental deployment of FANS vs. conventional ureteral sheaths. This comparative design was implemented to rigorously assess hypothesized improvements in safety and efficacy profiles attributable to the novel device. The analysis revealed that the group using the FANS had a higher immediate SFR, which was statistically significant, consistent with the conclusions of Zhu et al (15). The reason is that while the conventional ureteral access sheath RIRS combined with Needle-perc can completely fragment stones, it can only retrieve larger fragments with a stone basket, leaving the stone debris to be expelled by the patient postoperatively. In patients with anatomical abnormalities of the renal lower calyx, influenced by factors such as the infundibulopelvic angle (IPA), the lower calyx neck width (IW), and stone composition (16), up to 54.4% of residual fragments cannot be cleared in the first phase (17). The RIRS with FANS has a greater deflection angle compared to the RIRS with a conventional ureteral access sheath, making it easier to reach the target calyx for stone clearance. Previously, for patients with IPA < 30°, changing body positions to alter angles to assist RIRS in entering the renal lower calyx was employed; however, this method is not only complex but also increases surgical time, and the stone expulsion rate cannot be guaranteed. Upon data collection, it was observed that some patients had IPA < 30° in conjunction with IW < 5 mm or a lower calyx neck length (IL) ≥ 3 cm. Even whenthe RIRS with FANS cannot enter the target calyx for stone clearance, the negative pressure can still remove the stone debris from the target calyx after Needle-perc-assisted fragmentation, thereby improving the immediate SFR in patients withvarious anatomical abnormalities of the lower calyceal (18).

Upon analyzing the data, we found a statistically significant difference in operative times between the FANS group and the conventional ureteral access sheath group, with the former demonstrating shorter operative time. This difference may be attributed to the following three aspects: First, the clarity of the surgical field is a key factor affecting operative efficiency. The FANS group, due to the presence of negative pressure suction, can more effectively maintain the clarity of the surgical field (19), thereby reducing operational difficulties and time wastage caused by blurred vision, thus shortening the operative time. Second, RIRS with a conventional ureteral access sheath has limitations when operating on the renal lower calyx, ureteral wall, or recessed anatomical areas, making it difficult to act directly on these areas. This leads to constant adjustments of the RIRS position and angle during surgery, increasing the complexity and time consumption of the procedure. In contrast, the RIRS with FANS has a greater bending angle, allowing it to reach the target calyx more flexibly, reducing the number of adjustments needed during surgery, and consequently shortening the operative time. Lastly, although we have not yet quantified the size distribution of stone fragments during the operation, based on surgical experience, the use of FANS allows for the aspiration of stone powder (approximately 1 mm debris) during lithotripsy, eliminating the need to withdraw the scope or use a stone basket to retrieve stone fragments. The statistically significant difference in operative time between the two groups in our study results further supports that this approach reduces the time required for stone removal operations. In contrast, the FANS only needs to aspirate stone powder, making the operation simpler and further reducing operative time. Additionally, one case of pulmonary embolism was found in the conventional ureteral access sheath group, with a surgery duration of 144 min, while no similar postoperative complications were observed in the FANS group. Previous studies have reported (20) that operative duration is a risk factor for postoperative venous thrombosis, emphasizing that the longer the surgery, the higher the risk of related complications. We re-examined the patient's medical record and orders. The patient's positioning and fluid administration were comparable to those of other patients. Postoperatively, the patient experienced pain in the left lower limb and underwent ultrasound examination, which revealed a venous thrombus. A contrast-enhanced chest CT scan suggested the possibility of pulmonary embolism. A vascular surgery consultation was conducted, and low-molecular-weight heparin was administered for treatment. The patient was also instructed to remain in absolute bed rest to prevent further dislodgement of the lower limb venous thrombus, which could lead to massive pulmonary embolism. Considering that the patient's other perioperative variables did not differ significantly compared with those of the other patients, and the only difference was the longer operative time, we concluded that the extended operative time might have contributed to the formation of postoperative lower limb thrombosis, which subsequently led to pulmonary embolism. Therefore, the shorter operative time of the FANS group helps to reduce the risk of related complications due to longer surgery durations, enhancing the safety of the procedure.

Concurrently, we observed a notable disparity in secondary intervention requirements: one case in the FANS cohort vs. four cases in the conventional sheath group. This outcome suggests that FANS deployment may potentially reduce hospital readmissions and lower associated healthcare expenditures.

In the past, the effectiveness of laser lithotripsy was often judged by the SFR at one month postoperatively, with less attention paid to the immediate SFR. However, with the development of minimally invasive treatment techniques and the increased use of postoperative stents, the risk and concern regarding ureteral steinstrasse formation have increased (21). A higher number of residual stones and longer indwelling times can increase the risk of postoperative hematuria, renal colic, and urinary system infections, increasing the difficulty of treatment and patient suffering. If steinstrasse is not treated in time, it can lead to infection (22), and in severe cases, it can be life-threatening (23). Although the placement of a D-J stent after lithotripsy can facilitate drainage, residual stone expulsion, and prevent stricture, it also inhibits ureteral peristalsis. When there is a large amount of stone powder, the risk of ureteral steinstrasse is significantly increased. Vaddi (24) reported complications of ureteral steinstrasse after RIRIS. Currently, there are various treatment methods for steinstrasse, but powdered stones can easily block the ureteral stent, affecting the safety of the surgery. The FANS can improve the immediate SFR, prevent the formation of steinstrasse, ensure postoperative outcomes, and reduce complications.

The management of low-burden lower calyceal stones remains a persistent clinical challenge. To address this, our center pioneered the integrated innovation of Needle-perc and FANS technologies for patients with <2 cm stones in anatomically unfavorable lower calyces. Current outcomes validate that this combined approach establishes a novel therapeutic framework for this specific patient subset.

This study has limitations. First, the retrospective design inherently introduces selection bias—for instance, surgeons' preferential use of FANS in cases deemed anatomically amenable. Second, constrained timeframes resulted in limited post-matching sample size (*n* = 33 per group), insufficient follow-up duration (<6 months), and undocumented lithotripsy times in surgical records—limitations to be addressed in future investigations. Finally, while combined KUB/CT imaging assessed 1-month stone-free rates (SFR), forthcoming prospective studies will implement standardized CT follow-up protocols as the gold standard to enhance methodological rigor.

## Conclusion

In the NAES procedure, compared with conventional ureteral access sheath, the FANS ensures safety while also demonstrating greater effectiveness for treating kidney stones in patients with unfavorable renal lower calyx anatomy of less than 2 cm.

## Data Availability

The original contributions presented in the study are included in the article/Supplementary Material, further inquiries can be directed to the corresponding author.
